# Advertisements

**Published:** 1886-01-20

**Authors:** 


ADVERTISEMENTS.—Read our Advertisements. They are interesting and often instructive to the reader. The index on our first cover page will show you where to find them. The following are new—first entered with the present month : New York Pharmaceutical Company, or Viburnum Compound ; Hall & Bailey, or Anderson's Vaginal Capsules ; Sharp & Dohme, Solid and Fluid Extracts, etc.

---

THE several children bitten by a mad dog in New Jersey some weeks ago were sent to M. Pasteur, of Paris, to be inoculated for hydrophobia. They embarked on the steamer Canada, on the 9th of December, a few days subsequent to the time of the biting. They arrived at Havre on time, and went directly on to Paris, reaching there December 22. Pasteur had been telegraphed and was on the lookout for the party ; he received them kindly, and inoculated the children on the very night of their arrival, which was followed by a series of inoculations ; after which they were returned to their homes, and are now considered as thoroughly protected against hydrophobia.

DR. DUJARDE BEAUMETZ reported to the Hygienic Society of Paris a case of spontaneous hydrophobia, having all the characteristic symptoms, and confirmed by the fact that rabbits inoculated from the patient took the disease. We opine that, although it is said the patient had never been bitten by a dog, he had in some way been inoculated with the virus from a rabid animal.

THE PEPTOGENIC MILK POWDERS, prepared by that reliable and popular house, Fairchild Brothers & Foster, of New York, is a superior preparation in cases where the babe is deprived of its mother's milk. It is one which we feel safe in recommending to the profession, having tested it in our practice and found excellent results. The preparation is described in a communication signed by Albert Leeds, Ph. D., Chemist, as found in this journal, which the practitioner will do well to examine.

